# LYZ2-SH3b as a novel and efficient enzybiotic against methicillin-resistant *Staphylococcus aureus*

**DOI:** 10.1186/s12866-023-03002-9

**Published:** 2023-09-13

**Authors:** Marzieh Asadi, Mortaza Taheri-Anganeh, Maryam Ranjbar, Seyyed Hossein Khatami, Amir Maleksabet, Zohreh Mostafavi-Pour, Younes Ghasemi, Abdolkhalegh Keshavarzi, Amir Savardashtaki

**Affiliations:** 1 Department of Medical Biotechnology, School of Advanced Medical Sciences and Technologies, Shiraz University of Medical Sciences Shiraz, Shiraz, Iran; 2 Student Research Committee, Shiraz University of Medical Sciences, Shiraz, Iran; 3 Cellular and Molecular Research Center, Cellular and Molecular Medicine Research Institute, Urmia University of Medical Sciences, Urmia, Iran; 4 Department of Clinical Biochemistry, School of Medicine, Shahid Beheshti University of Medical Sciences, Tehran, Iran; 5 Department of Medical Biotechnology, School of Advanced Technologies in Medicine, Mazandaran University of Medical Sciences, Sari, Iran; 6 Recombinant Protein Laboratory, Department of Biochemistry, School of Medicine, Shiraz University of Medical Sciences, Shiraz, Iran; 7 Pharmaceutical Sciences Research Center, Shiraz University of Medical Science, Shiraz, Iran; 8 Burn and Wound Healing Research Center, Shiraz University of Medical Science, Shiraz, Iran; 9 Infertility Research Center, Shiraz University of Medical Sciences, Shiraz, Iran

**Keywords:** *Staphylococcus aureus*, SH3b domain, Enzybiotic, MRSA, Exolysin

## Abstract

**Background:**

Enzybiotics are promising alternatives to conventional antibiotics for drug-resistant infections. Exolysins, as a class of enzybiotics, show antibacterial effects against methicillin-resistant *Staphylococcus aureus* (MRSA). This study evaluated a novel exolysin containing an SH3b domain for its antibacterial activity against MRSA.

**Methods:**

This study designed a chimeric exolysin by fusing the Cell-binding domain (SH3b) from Lysostaphin with the lytic domain (LYZ2) from the gp61 enzyme. Subsequently, LYZ2-SH3b was cloned and expressed in *Escherichia coli (E. coli).* Finally, the antibacterial effects of LYZ2-SH3b compared with LYZ2 and vancomycin against reference and clinical isolates of MRSA were measured using the disc diffusion method, the minimal inhibitory concentration (MIC), and the minimal bactericidal concentration (MBC) assays.

**Results:**

Analysis of bioinformatics showed that LYZ2-SH3b was stable, soluble, and non-allergenic. Protein purification was performed with a 0.8 mg/ml yield for LYZ2-SH3b. The plate lysis assay results indicated that, at the same concentrations, LYZ2-SH3b has a more inhibitory effect than LYZ2. The MICs of LYZ2 were 4 µg/mL (ATCC 43,300) and 8 µg/mL (clinical isolate ST239), whereas, for LYZ2-SH3b, they were 2 µg/mL (ATCC 43,300) and 4 µg/mL (clinical isolate ST239). This suggests a higher efficiency of LYZ2-SH3b compared to LYZ2. Furthermore, the MBCs of LYZ2 were 4 µg/mL (ATCC 43,300) and 8 µg/mL (clinical isolate ST239), whereas, for LYZ2-SH3b, they were 2 µg/mL (ATCC 43,300) and 4 µg/mL (clinical isolate ST239), thus confirming the superior lytic activity of LYZ2-SH3b over LYZ2.

**Conclusions:**

The study suggests that phage endolysins, such as LYZ2-SH3b, may represent a promising new approach to treating MRSA infections, particularly in cases where antibiotic resistance is a concern. But further studies are needed.

**Supplementary Information:**

The online version contains supplementary material available at 10.1186/s12866-023-03002-9.

## Background

Nowadays, antibiotic resistance causes numerous problems for the medical community in treating bacterial diseases. This problem is even more significant when it comes to *staphylococci*, which have a high level of drug resistance than most other bacteria [1].

*Staphylococcus aureus* (*S. aureus*) is a gram-positive bacterium found as natural microflora in human and animal bodies. These bacteria can cause skin infections, food poisoning, dangerous shocks, and autoimmune disorders under special conditions such as stress, viral diseases, tissue injury, or any factor weakening the immune system [2, 3]. Most strains of *S. aureus* have become resistant to antibiotics, including penicillin, methicillin, and vancomycin. Moreover, like other drugs, antibiotics have side effects, including allergic reactions, toxicity to various organs (e.g., hepatic and renal toxicity), and an inhibitory impact on natural flora. Therefore, finding materials with antibacterial properties is one of the basic needs in the battle against antibiotic-resistant *S. aureus* [4].

Researchers have focused on phage therapy due to widespread antibiotic use. Most *S. aureus* phages belong to the *Caudovirales* order and are strictly lytic [5]. However, the difficulty of purifying phages and transferring toxin genes from phages to bacteria has limited the application of this therapeutic method [6, 7]. Due to the challenges mentioned above, in the last decade, researchers have paid special attention to the use of peptidoglycan hydrolases (PGHs) generated by bacteriophages rather than the direct use of phages. Numerous investigations have revealed that these enzymes are antibacterial against gram-positive pathogens [8–14]. Through the specific destruction of the PG layer in the bacterial cell wall, these enzymes cause bacteria lysis and death. Hence, these antibiotic-like enzymes, known as enzybiotics, can serve as an alternative to antibiotics in the face of resistance [1, 6]. The PGHs produced by phages participate in the infection cycle by degrading peptidoglycan; therefore, most phages can produce these enzymes [1]. PGHs fall into two categories: endolysins and exolysins. Endolysins are enzymes manufactured by phages after replication to break the PG layer and exit the bacterial host. These enzymes often have an N-terminal catalytic domain and a C-terminal cell wall binding domain (CBD) in their structure (1). The other category of PGHs includes exolysins, which are often referred to by other names such as tail-associated lysins (TAL), tail-associated muralytic enzymes (TAME), or virion-associated peptidoglycan hydrolases (VAPGH). By destroying the PG layer, these enzymes facilitate the entrance of phage DNA into bacterial cells during an infection. In contrast to endolysins, these enzymes have one or two catalytic domains for destroying the PG layer and lack the wall binding domain [15].

The enzyme gp61, a type of exolysin, is synthesized by the phage ΦMR11. Gp61 has an N-terminal CHAP (Cysteine, histidine-dependent amidohydrolases/peptidases) domain and a C-terminal LYZ2 domain, which performs the enzyme’s catalytic activity. Recent structural investigations of this protein indicate that the LYZ2 domain has much greater catalytic activity than the other domains of this enzyme. Furthermore, this enzyme lacks a cell wall binding domain [16]. Bacteriocin lysostaphin is an enzyme produced by *Staphylococcus simulans*. Structure analyses show this enzyme has a C-terminal CBD named SH3b and its catalytic domain. This domain explicitly identifies the PG layer of *S. aureus*. Recent research indicates that using the SH3b domain of Lysostaphin to produce engineered chimeric enzybiotics significantly increases their activity and efficiency against the pathogen *S. aureus* [6, 17].

Numerous studies have been conducted on the role and production of chimeric endolysins in fighting *S. aureus* bacteria [3, 8, 9, 18]. Meanwhile, few studies have focused on using exolysins as enzybiotic agents comparable to endolysins. Also, Modifications of phage lytic enzymes lead to the creation of ideal enzybiotics that have high activity and desirable properties such as solubility, thermostability, high specificity, or broad-spectrum activity depending on the target infection, are protease-resistant, and should be economical to produce. Hence, the engineering and design of new enzybiotics offer countless opportunities for creating a popular and commercially viable product. Although many cases of engineered enzybiotics have been produced and have shown successful antimicrobial effects, it is crucial to identify which ones possess appropriate properties and is ideal for commercial production [19].

Phage-lytic peptidoglycan hydrolases, such as endolysins and exolysins, are antibacterial against gram-positive pathogens. However, this enzyme is not specific for identifying *S. aureus.* SH3b is the cell-binding domain of the bacteriocin lysostaphin, which specifically targets the peptidoglycan layer of *S. aureus* [1]. Therefore, this study aimed to use the combination of LYZ2 exolysin and SH3b to produce a newly engineered enzybiotic that specifically identifies the PG layer of *S. aureus.*

## Results

### Analysis of bioinformatics

**Physicochemical Properties, Solubility, and Allergenicity**.

Table 1 presents the physicochemical properties of LYZ2-SH3b. According to ProtParam analysis, the MW and theoretical pI of the LYZ2-SH3b were 36 kDa and 9.85. The instability and aliphatic indexes were determined to be 28.31 and 58.98, respectively. Additionally, the GRAVY value was − 0.623. The protein’s solubility was calculated as 0.517 using SoLpro servers, indicating that LYZ2-SH3b was predicted to be a soluble protein. According to the AllerTOP results, LYZ2-SH3b was likely to be a non-allergen (Table 1).

**Table 1:** Physicochemical properties, Solubility and Allergenicity of LYZ2-SH3b.

Analysis of the Physicochemical Properties								Solubility	Allergenicity
**LYZ2-SH3b**	ProtParam tool (EXPASY server)								
	Amino Acid Length	MW (kDa)	pI	Instability index	Aliphatic Index	GRAVY	Half-life	SOLpro	AllerTOP
	321	36	9.85	28.31	58.98	-0.623	> 10 h (Escherichia coli, in vivo)	Soluble (0.517)	Non-Allergen

**Abbreviation**: MW: Molecular weight; pI: Isoelectric point; GRAVY: Grand average of hydropathicity

### Gene optimization

A wide variety of factors regulate and influence gene expression levels. The optimumGeneTM algorithm considers as many of them as possible, producing a single gene that can reach the highest possible expression level. In this case, the native gene employs tandem rare codons that can decrease translation efficiency or even disengage from the translational machinery. This algorithm changed the codon usage bias in *E. coli* by upgrading the CAI from 0.50 to 0.96. A CAI of 1.0 is ideal, and a CAI of *≥* 0.8 is considered appropriate for expression in the host organism (Sup 1 A). GC content and unfavorable peaks have been optimized to prolong mRNA half-life. The GC content changed from 44.29 to 54.60. The ideal percentage range of GC content is between 30 and 70% (Sup 1B). FOP of 91–100, 81–90, and 71–80 in the gene after optimization were 84,6 and 8%, respectively. The value of 100 is set for the codon with the highest usage frequency for a given amino acid in the desired expression organism (Sup 1 C).

### Construction, expression, and purification of the recombinant LYZ2-SH3b and LYZ2

The optimized DNA sequences of LYZ2-SH3b (962 bp) and LYZ2 (695 bp) were correctly cloned into competent *E. coli* DH5α cells. Protein purification was performed using a Ni-NTA column yielding 0.8 mg/ml for LYZ2-SH3b and 0.5 µg/ml for LYZ2. The purity and molecular weight (MW) of the proteins (LYZ2-SH3b: 36 kDa and LYZ2: 26 kDa) were confirmed through SDS-PAGE analysis (Sup 2 A-B). Furthermore, western blot analysis authenticated all the proteins (Sup 2 C).

### Microbiological assays

We assessed the lytic activities of the LYZ2 and LYZ2-SH3b enzymes against MRSA ATCC 43,300 and clinical isolate ST239 through plate lysis, MIC, and MBC assays. Plate lysis assay results are detailed in Table 2 and Sup 3. This assay revealed the diameters of the clear zone around the LYZ2 and LYZ2-SH3b spots at various concentrations. At the same concentrations, LYZ2-SH3b exhibited a more substantial bactericidal effect than LYZ2 for both strains. The MICs of LYZ2 were found to be 4 µg/mL (ATCC 43,300) and 8 µg/mL (clinical isolate ST239), whereas, for LYZ2-SH3b, they were 2 µg/mL (ATCC 43,300) and 4 µg/mL (clinical isolate ST239). This suggests a higher efficiency of LYZ2-SH3b than LYZ2 (Table 3 and Sup 4). Furthermore, the MBCs of LYZ2 were 4 µg/mL (ATCC 43,300) and 8 µg/mL (clinical isolate ST239), whereas, for LYZ2-SH3b, they were 2 µg/mL (ATCC 43,300) and 4 µg/mL (clinical isolate ST239), thus confirming the superior lytic activity of LYZ2-SH3b over LYZ2 (Table 3 and Sup 5). The results indicated that the antibacterial activity of vancomycin in disc diffusion, MIC, and MBC assays, when compared with LYZ2-SH3b at the same concentrations, is remarkably higher (Tables 2 and 3 and Sup 3–5).

**Table 2:** Disc diffusion diameter for various concentrations of LYZ2, LYZ2-SH3b, and vancomycin

Disc diffusion diameter (mm)						
Compound	MRSA: clinical isolate ST239			MRSA: ATCC 43,300		
	50 µg/ml	5 µg/ml	0.5 µg/ml	50 µg/ml	5 µg/ml	0.5 µg/ml
**LYZ2**	11 ± 0.3	5 ± 0.4	0	15 ± 0.2	10 ± 0.4	0
**LYZ2-SH3b**	16 ± 0.5	10 ± 0.2	0	19 ± 0.5	12 ± 0.3	0
**Vancomycin**	20 ± 0.2	14 ± 0.4	5 ± 0.3	22 ± 0.3	18 ± 0.4	8 ± 0.3

**Table 3:** The MIC and MBC for various concentrations of LYZ2-SH3b, LYZ2, and vancomycin;

Compound	MRSA: ATCC 43,300		MRSA: clinical isolate ST239	
	MIC (µg/ml)	MBC (µg/ml)	MIC (µg/ml)	MBC (µg/ml)
**LYZ2**	≥ 4	4	≥ 8	8
**LYZ2-SH3b**	≥ 2	2	≥ 4	4
**Vancomycin**	≥ 0.5	0.5	≥ 0.5	0.5

## Discussion

Antibiotic resistance in clinical settings is a growing concern, and novel therapeutic options are needed beyond conventional antibiotics [20]. Additionally, it’s crucial to consider the antibiotic’s ability to target specific bacterial strains or avoid resistance mechanisms [21, 22]. In this study, we designed and synthesized a chimeric enzyme (LYZ2-SH3b) by fusing the catalytic LYZ2 domain of gp61 with the SH3b binding domain of Lysostaphin. SH3b domain specifically identifies *S. aureus’* PG layer. Following, we evaluated the antibacterial effects of LYZ2-SH3b compared with LYZ2 and vancomycin. This represents the first report on designing and synthesizing this specific chimeric enzyme. The results of this study suggest that LYZ2-SH3b may represent a promising new approach to treating MRSA infections.

We predicted its physicochemical properties after designing the chimeric enzyme (LYZ2-SH3b) (Table 1). Our findings reveal that LYZ2-SH3b is a novel enzybiotic with proper activity and efficacy against MRSA. The GRAVY score (-0.623) indicates that the designed chimeric enzyme is hydrophilic and capable of interacting with water molecules in solution, implying its solubility. This result was confirmed by the SOLpro server. Moreover, the aliphatic index (58.98) demonstrates that the chimeric enzyme possesses adequate aliphatic amino acids, potentially enhancing its thermostability. The instability index indicates that this protein is stable (28.31). Additionally, our results showed that LYZ2-SH3b is a probable non-allergen. The properties mentioned, including hydrophilicity, solubility, thermostability, stability, and non-allergenicity, are all essential factors to consider when designing a protein for antibiotic production.

After expressing and purifying LYZ2-SH3b, its antibacterial activity against MRSA was compared with that of LYZ2 using various methods, including disc diffusion, MIC, and MBC. The plate lysis assay showed that at similar concentrations, the diameter of the clear zone was not the same for LYZ2-SH3b and LYZ2 (Table 2 and Sup 3). The MIC and MBC determinations also showed a difference in the efficacy between LYZ-SH3b and LYZ2 (Table 3 and Sup 3–5). These findings indicate that the LYZ2-SH3b protein exhibits distinct antimicrobial activity compared to LYZ2 alone. These promising results suggest that the chimeric LYZ2-SH3b protein holds significant potential as an effective antimicrobial agent against MRSA.

SH3b cell-binding domain accounts for the heightened lytic activity of LYZ2-SH3b against MRSA compared to LYZ2. SH3b domain enhances the catalytic domain’s ability to locate and bind to its target bonds within the peptidoglycan layer of the bacterial cell wall [23, 24]. The beta-barrel structure of the SH3b domain enables the formation of a binding cleft capable of recognizing and attaching to specific amino acid residues within the peptidoglycan layer. This binding is essential for the efficient cleavage of peptidoglycan bonds by the catalytic domain of the enzyme. Consequently, adding the SH3b domain to LYZ2 likely enhances its efficacy by increasing its binding ability to the bacterial cell wall and targeting the peptidoglycan layer for degradation [24]. Furthermore, the SH3b domain also contributes to the overall stability of the enzyme by acting as a scaffold that helps maintain the proper conformation of the protein. The beta-barrel structure of the SH3b domain provides a sturdy framework for the entire enzyme structure, which can help prevent protein denaturation or breakdown [24].

Numerous studies have substantiated the hypothesis that enzybiotics can effectively combat MRSA. For instance, Rubio et al. [25] created three chimeric enzymes by merging the SH3b binding domain of Lysostaphin with the phage enzyme HydH5. They then evaluated their efficacy against MRSA. The resulting chimeric enzymes were named HydH5SH3b, HydH5Lyso, and CHAPSH3b. The results revealed that these chimeric enzymes exhibited higher antimicrobial activity against MRSA than HydH5 alone. The authors suggested these chimeric enzymes could serve as alternative antibiotics for treating MRSA infections. In another study, the SH3b binding domain of Lysostaphin was combined with the catalytic CHAP domain of Lys16, creating a chimeric enzyme known as p128. This chimeric enzyme demonstrated significant antimicrobial activity against *S. aureus* [26]. Additionally, it was noted that removing the SH3b domain from the Twort phage endolysin led to a roughly tenfold reduction in its enzyme activity [27]. By fusing the CHAP phage endolysin with the cell-binding domain of lysostaph (SH3b), a chimeric enzyme called CHAP-SH3b was developed, which displayed increased effectiveness against *S. aureus* [8].

In the present study, we compared the antibacterial effects of the LYZ2-SH3b protein with those of the vancomycin antibiotic using disc diffusion, MIC, and MBC assays. While LYZ2-SH3b exhibited bactericidal activity against MRSA, vancomycin proved more effective at bactericidal activity. This observation suggests that LYZ2-SH3b could hold potential as an alternative or supplementary antimicrobial agent but might not match the potency of conventional antibiotics such as vancomycin (Table 3 and Sup 3–5). Vancomycin is a potent antibiotic effective against Gram-positive bacteria, including *S. aureus*. Its action involves binding to the peptidoglycan layer of the bacterial cell wall, hindering cell wall synthesis and resulting in bacterial death [28].

In contrast, LYZ2-SH3b functions as an enzybiotic, targeting the peptidoglycan layer by enzymatically cleaving the bonds between sugar and amino acid subunits within the peptidoglycan. While this mechanism can be effective against bacteria, it might not possess the same potency as vancomycin’s multi-faceted action, which targets various aspects of cell wall synthesis [1]. This study indicates that while LYZ2-SH3b exhibits bactericidal activity against *S. aureus*, its effectiveness isn’t as pronounced as vancomycin. Further research could be necessary to optimize the enzyme’s activity and ascertain its potential as an antibiotic alternative for treating bacterial infections. Combining LYZ2-SH3b with other enzybiotics targeting distinct components of the bacterial cell wall may heighten its potency against *S. aureus* and other Gram-positive bacteria.

Nevertheless, further research is required to pinpoint the optimal combinations of enzybiotics and assess their potential for clinical applications. Some studies have investigated combination therapy using enzybiotics for treating bacterial infections. For instance, a study investigated the synergistic action of the bacteriophage phiIPLA-RODI and the lytic protein CHAPSH3b against *S. aureus* biofilms. The research revealed that the combination of phiIPLA-RODI and CHAPSH3b exhibited increased efficacy in reducing *S. aureus* biofilm biomass compared to individual agent treatments [29]. Although LYZ2-SH3b might not possess the same potency as antibiotics like vancomycin, its utility as an enzybiotic offers several advantages for tackling bacterial infections. LYZ2-SH3b tends to be more precise in targeting bacterial cells, lowering the risk of off-target effects on other body cells. Furthermore, they are less likely to promote antibiotic resistance in bacteria. However, further investigation is imperative to optimize enzybiotic safety and effectiveness and ascertain their potential for clinical use [30].

This study suggests that phage exolysins, such as LYZ2-SH3b, could introduce a promising new approach to treating MRSA infections, especially in scenarios where concerns about antibiotic resistance arise. Nevertheless, additional studies are necessary to refine the dosing and delivery strategies for these enzybiotics and assess their potential for inducing resistance or cross-reactivity with other bacterial species. A comprehensive review of the literature reveals numerous studies focused on the antibacterial activity of enzybiotics against *S. aureus*, with most of these investigations conducted in laboratory culture media [2, 3, 8, 18, 29]. Therefore, it is recommended that the results generated in this study and their implications undergo evaluation using animal models and during clinical trial phases. Additionally, the combined effect with other enzybiotics should also be considered.

In conclusion, antibiotic resistance is a concern in clinical settings, and novel therapeutic options are needed beyond conventional antibiotics. In this study, we designed and synthesized a chimeric enzybiotic, LYZ2-SH3b, demonstrating proper activity and efficacy against MRSA. Adding the SH3b cell-binding domain to LYZ2 increased its effectiveness against MRSA, suggesting that enzybiotics like LYZ2-SH3b may have potential as alternative or complementary antimicrobial agents. Although LYZ2-SH3b is not as potent as the antibiotic vancomycin, it presents several advantages, including its ability to specifically target bacterial cells and its reduced potential for promoting antibiotic resistance. Further research is needed to optimize the safety and efficacy of enzybiotics and determine their potential for use in clinical applications, including measuring their effect in combination with other enzybiotics. Overall, the study suggests that phage exolysins, such as LYZ2-SH3b, represent a promising new approach to treating MRSA infections, particularly in cases where antibiotic resistance is a concern.

## Materials and methods

### Bacterial strains, media, and cultivation conditions

Two strains of MRSA were employed to determine antibacterial activity: (i) the reference strain (ATCC 43,300) and (ii) a clinical isolate, ST239. The *E. coli* strains DH5α and BL21 (DE3) (Novagen, USA) were utilized for plasmid cloning and expressing the chimeric enzymes (LYZ2-SH3b). Mueller-Hinton broth and agar (MHB and MHA; Beijing Land Bridge Technology Co., Ltd., China) were utilized for bacterial growth. Bacteria were cultured overnight at 37 °C.

### Sequence Retrieval and chimeric protein Designing

**Fig. 1:**
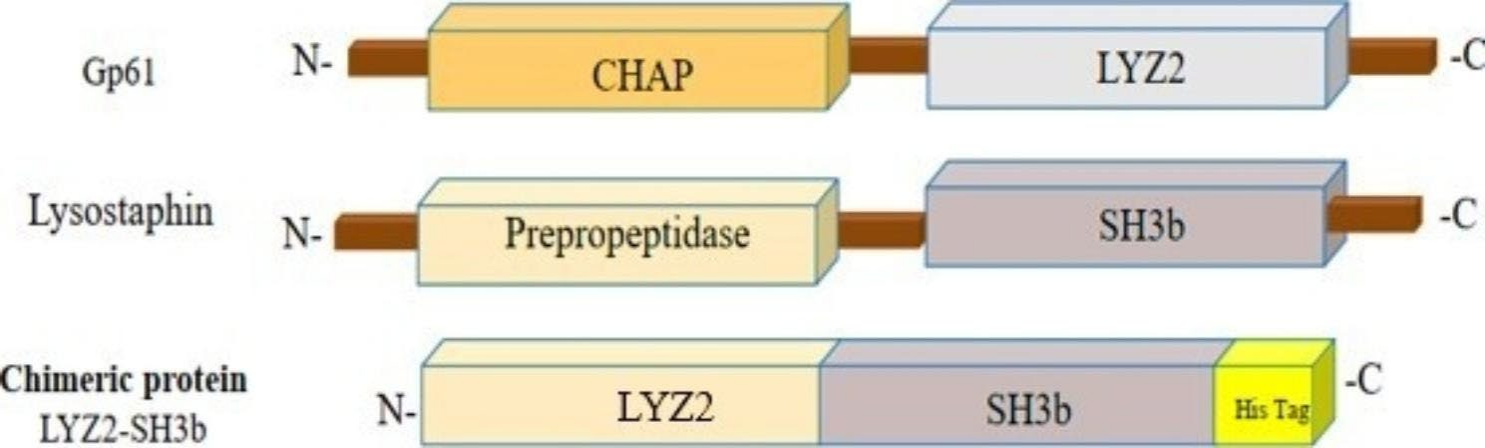
Schematic representation of gp61, Lysostaphin, and LYZ2-SH3b domains. The enzyme gp61, as a type of exolysin, is synthesized by the phage ΦMR11. Gp61 has two different catalytic domains CHAP (Cysteine, histidine-dependent amidohydrolases/peptidases) and an LYZ2 domain. The bacteriocin lysostaphin is an enzyme produced by *Staphylococcus simulans*. This enzyme has a C-terminal cell-binding domain (SH3b) and a catalytic domain (Prepropeptidase). LYZ2-SH3b is the chimeric protein generated for this study, and it is composed of the LYZ2 domain from gp61 and the SH3b cell-binding domain from Lysostaphin

We obtained the amino acid sequences of the LYZ2 (Accession No: AB360386) and SH3b (Accession No: M15686) domains from the UniProt database (https://www.uniprot.org/) in FASTA format. Subsequently, these sequences were directly joined without linkers (Fig. 1).

### Analysis of Bioinformatics

#### Evaluation of Physicochemical Properties, solubility, and allergenicity

The ProtParam tool on the ExPASy server (https://web.expasy.org/protparam/) was utilized to evaluate the physicochemical properties of LYZ2-SH3b. These properties include the instability index, theoretical PI, amino acid composition, molecular weight (MW), aliphatic index, estimated half-life, and the grand average of hydropathicity (GRAVY). The solubility of LTZ2-SH3b in the *E. coli* host was predicted using the SOLpro server (https://scratch.proteomics.ics.uci.edu/). Furthermore, allergenicity was checked by the AllerTOP 2.0 server (https://www.ddg-pharmfac.net/AllerTOP/).

### Back translation and gene optimization

The sequence (LYZ2-SH3b) was back-translated using the EMBOSS Backtranseq server (https://www.ebi.ac.uk/Tools/st/emboss_backtranseq/). To attain a significant increase in LYZ2-SH3b expression within *E. coli*, gene optimization was carried out employing the OptimumGeneTM Optimization algorithm. This process involved the use of the algorithm available at (https://www.genscript.com/tools/rare-codon-analysis). Various sequence factors were evaluated, such as GC content, codon adaptation index (CAI), frequency of optimal codons (FOP), CIS-acting elements, and repeat sequences.

### Plasmids construction and cloning

DNA fragments for LYZ2-SH3b were synthesized (Biomatik, Canada) and inserted into the pET-28a (+) expression vector (Novagen, Madison, WI, USA) at the *EcoRI/XhoI* restriction site. Subsequently, DNA fragments for LYZ2 were amplified from LYZ2-SH3b through PCR, employing forward and reverse primers (Table 4) from the (pET-28a-LYZ2-SH3b) vector. The amplified product was cloned into the *NcoI* and *XhoI* restriction sites of pET-28a (+). Chemically competent *E. coli* DH5α cells (Novagen, USA) were transformed using a heat shock protocol with the recombinant constructs (pET-LYZ2-SH3b and pET-LYZ2). The plasmids were extracted from transformants using GeneAll Plasmid Miniprep Kit (GeneAll, Korea), and the accuracy of the cloning was confirmed by nucleic acid sequencing.

**Table 4:** Primers used for amplification of LYZ2.

Primers	Length	Sequences
LYZ2 Forward primer	5′-AAAACCATGGGCGTGACCGAGACCAGC-3′	27 nt
LYZ2 Reverse primer	5′-AAAACTCGAGTTAGTGGTGGTGGTGGTGGTG TTTGTACTTATCACGGGTAAAG-3′	53 nt

### Expression and purification of LYZ2-SH3b and LYZ2

*E. coli* BL21 (DE3) (Novagen, USA) chemically competent cells were transformed with recombinant plasmids (pET-LYZ2-SH3b and pET-LYZ2) and grown at 37 ºC in 100 mL of Luria Broth (LB) medium containing 100 µg/mL kanamycin. To induce protein expression, after reaching the cell density to an OD_600_ of 0.5, 1 mM isopropyl-b D-thiogalactopyranoside (IPTG) was added to the cultures, which were incubated for 6 h at 37 ºC (160 rpm). Cells were then harvested by centrifugation at 6000 ×g at 4 °C for 20 min. Bacterial pellets were resuspended and homogenized in 5 ml lysis buffer (50 mM Na_2_HPO_4_, 300 mM NaCl, 10 Mm imidazole; pH = 8) for 45 min at 4 °C. PMSF (Phenylmethylsulfonyl Fluoride) as a protease inhibitor was added in 1 mM concentration for inhibiting proteases. The pellets were sonicated to disrupt bacteria under ice conditions: ten cycles of sonication at 45% amplitude for 10 s and intervals of 10 s. The resulting lysates were then centrifuged at 13,000×g for 30 min at 4 °C. Supernatants were applied to Ni-NTA resin (Qiagen, Hilden, Germany) for 2 h at 4 °C. Subsequently, the resin was washed twice with 5mL wash buffer (50 mM NaH2PO4, 25 mM imidazole, 300 mM NaCl; PH = 8). The specifically bound protein was washed out of the resin with 5 mL of elution buffer (50 mM NaH2PO4, 250 mM imidazole, 300 mM NaCl; PH = 8). Finally, the protein concentration was determined using the BCA protein assay kit (Thermo Fisher Scientific, USA).

### Sodium Dodecyl Sulfate-Poly Acrylamide Gel Electrophoresis (SDS–PAGE (and western blotting

The presence of recombinant proteins in the purified proteins (LYZ2-SH3b and LYZ2) was demonstrated through SDS-PAGE and confirmed by western blot analysis [31]. For SDS-PAGE analysis, the protein fractions (4 µg) were mixed with the loading buffer (0.5 M Tris–HCL (pH 6.8), SDS 10%, glycerol, bromophenol blue, and DTT), heated at 95 °C for 10 min, and electrophoresed on 12% polyacrylamide gel (95 V). The corresponding bands were visualized by Coomassie Brilliant Blue (Merck, Germany). For western blot analysis, The 0.2 μm nitrocellulose membrane was blotted with 50 ng of purified LYZ2-SH3b and LYZ2. The membranes were blocked in PBS-T (phosphate-buffered saline 1x supplemented with 0.15% Tween 20) and 5% non-fat skim milk for 90 min at 4 °C. After three rounds of washing with PBS-T, the membranes were incubated with Goat anti-6-His Tag Antibody HRP Conjugated (1:2000, absolute antibody, USA) for 2 h at room temperature. Finally, after three rounds of washing, the membranes were treated with 3, 3′-Diaminobenzidine (DAB) (Sigma-Aldrich, USA) to visualize the corresponding bands. Oxidized DAB generates a brown precipitate at the location of the HRP, which can be visualized with the naked eye.

### Antibacterial activity assays

The antibacterial activity of the purified enzymes (LYZ2 and LYZ2-SH3b) was measured through the disc diffusion method, minimal inhibitory concentration (MIC), and minimal bactericidal concertation (MBC) against two strains of MRSA (i) the reference strain (ATCC 43,300) and (ii) a clinical isolate ST239. All antibacterial activity assays were measured according to the guidelines of the CLSI (Clinical and Laboratory Standards Institute) [32]. In addition, a standard antibiotic (vancomycin) was included for antimicrobial activity comparison.

### Disc Diffusion Method

The antimicrobial activity of the purified enzymes (LYZ2 and LYZ2-SH3b) was assessed using the agar disc diffusion method [31]. Picking of a few clones of *S. aureus* (MRSA) from the MHA plate was resuspended in sterile distilled water to achieve a density of McFarland 0.5 (2 × 10^8^ CFU/ml). Subsequently, the purified enzymes were serially diluted 1:10 in PBS to achieve 50, 5, and 0.5 µg/ml concentrations. Then, 10 µl of each dilution was added to a blank antibiogram disk (6.4 mm) spotted onto a plate of *S .aureus*. The MHA plate was incubated at 37 °C overnight. The control plate was spotted with an antibiogram disc containing only buffer (PBS). The diameters of the inhibition zones formed by the enzymes were measured and compared.

### MIC and MBC assay

The MIC of each enzyme (LYZ2 and LYZ2-SH3b) was determined using microdilution in sterile 96-well plates [33]. Briefly, bacterial cells were cultured on Mueller-Hinton agar (MHA) and then diluted to a 5 × 105 CFU/ml concentration. To avoid the unspecific attachment of enzymes to the polystyrene surface of the plate, the wells of the microtiter plate were treated with a 0.5% solution of bovine serum albumin (BSA) and left to incubate for 2 h at 4°CSubsequently, fifty microliters of 1:2 serially diluted enzymes (LYZ2-SH3b and LYZ2) in PBS, were added to each well of the plates, followed by 50 µl of bacterial solution (0.5 McFarland standard). The plates were incubated overnight at 37 °C. Cultures without enzymes and PBS were positive and negative controls, respectively. After incubation, the MIC was determined to be the lowest concentration showing no visible growth. MBC was determined as the lowest enzyme concentration that killed 99.9% of *S. aureus* on agar plates after overnight incubation at 37 °C. As identified in the MIC determination experiment, an aliquot of 50 µl from each non-turbid well was spread on agar plates.

### Statistical analysis

All tests were carried out in duplicate. The data were represented as the mean ± standard deviation (SD). The experimental results were analyzed using a one-way analysis of variance (ANOVA) followed by Tukey’s post-hoc test for all pairwise comparisons. All statistical analyses were performed using the statistical package for social science version 19 (SPSS Inc., Chicago, Illinois, USA). In all cases, a p-value < 0.05 was established as the threshold for considering a result statistically significant.

### Electronic supplementary material

Below is the link to the electronic supplementary material.

Supplementary Material 1

## Data Availability

The amino acid sequences of LYZ2 and SH3b are accessible under the accession number AB360386 (https://www.uniprot.org/uniprotkb/A7VMY9/entry) and M15686 (https://www.uniprot.org/uniprotkb/P10547/entry) in UniProt database. The mentioned servers were used in this study; ProtParam tool at ExPASy Server (https://web.expasy.org/protparam/), SOLpro server (https://scratch.proteomics.ics.uci.edu/), AllerTOP server (https://www.ddg-pharmfac.net/AllerTOP/), EMBOSS Backtranseq server (https://www.ebi.ac.uk/Tools/st/emboss_backtranseq/) and gene optimization server (https://www.genscript.com/tools/rare-codon-analysis).

## Abbreviations

BSA: Bovine serum albumin
TAL: Tail-associated lysins
CBD: Cell binding domain
CHAP: Cysteine, histidine-dependent amidohydrolases/peptidases
DAB: 3, 3′-Diaminobenzidine
IPTG: Isopropyl-b Dthiogalactopyranoside
LYZ2: Lysozyme subfamily 2 domain
LB: Luria Broth
MHB: Mueller–Hinton Broth
MHA: Mueller–Hinton Agar
MRSA: Methicillin-resistant *Staphylococcus aureus*
MIC: Minimal inhibitory concentration
MBC: Minimal bactericidal concentration
PGH: Peptidoglycan hydrolases
PG: Peptidoglycan
PBS: Phosphate-buffered saline
SDS –PAGE: Sodium Dodecyl Sulfate-Poly Acrylamide Gel Electrophoresis
TAME: Tail-associated muralytic enzymes
VAPGH: Virion-associated peptidoglycan hydrolases
CAI: Codon Adaptation Index
CFD: Codon Frequency Distribution
PMSF: Phenylmethylsulfonyl Fluoride
