# Supplementary material for: LYZ2-SH3b as a novel and efficient enzybiotic against methicillin-resistant Staphylococcus aureus
Source: BMC Microbiol. 2023 Sep 13;23:257. doi: 10.1186/s12866-023-03002-9 (PMC10500863; doi:10.1186/s12866-023-03002-9)
Supplement: Supplementary file 1 — Supplementary Material 1 [file 12866_2023_3002_MOESM1_ESM.docx]

**
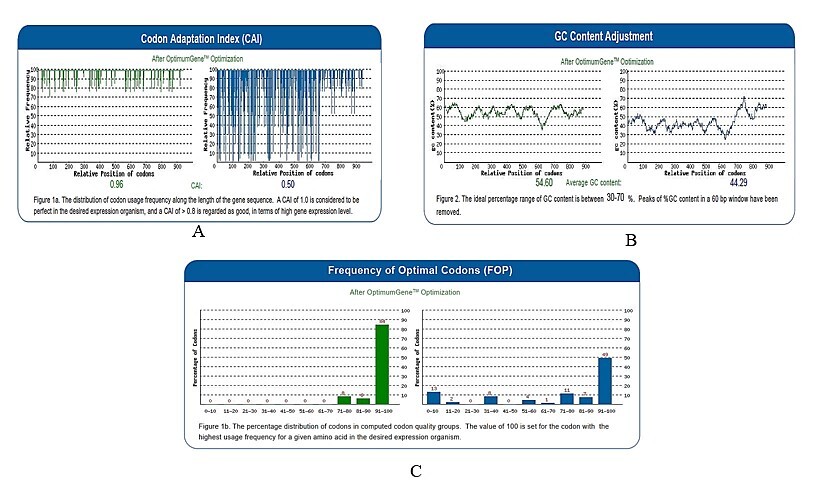
**

**Sup1:** Analysis of three parameters (CAI, GC content, and FOP) of gene-optimization for LYZ2-SH3b to overexpression in *E.coli*. CAI of the gene sequence changed from 0.50 to 0.96 (**A**), and GC content changed from 44.29 to 54.60 (**B**) after optimization. After optimization, the FOP of 91-100, 81-90, and 71-80 in the gene were 84, 6 and 8%, respectively (**C**). **Abbreviation: CAI:** Codon Adaption Index, **FOP:** Frequency of Optimal Codon.

**
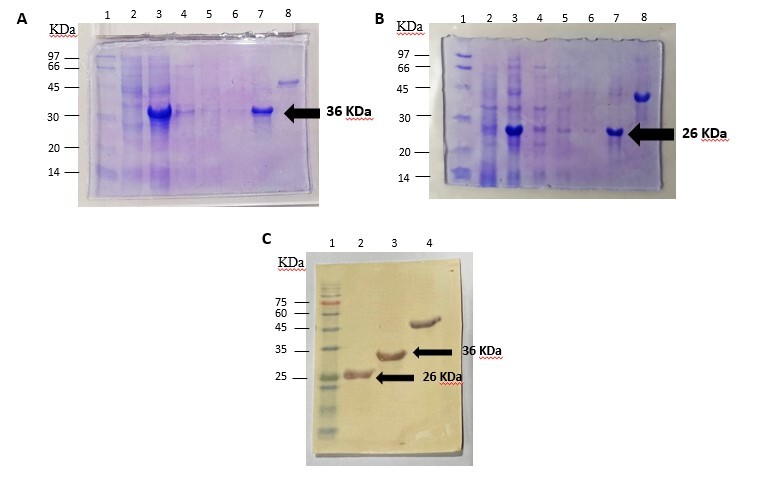
**

**Sup 2.** Recombinant variants of LYZ2-SH3b and LYZ2 proteins were analyzed using SDS-PAGE (A and B) and western blot analysis (C). **Sup 2A:** SDS-PAGE LYZ2-SH3b; line 1, protein size marker; Line 2, uninduced (indicating that the promoter is not activated and the protein is not being actively produced); line 3, induced (indicating that the promoter is activated by IPTG and the protein is actively being produced); line 4, prewash, lines 5 and 6, consecutive washes; line 7 final elution of LYZ2-SH3b (36 kDa) and line 8 is control protein (recombinant nucleocapsid (N) protein of SARS-CoV-2). **Sup 2B:** SDS-PAGE LYZ2; Line 1, protein size marker; Line 2, uninduced; Line 3, induced; Line 4, prewash; Lines 5 and 6, consecutive washes; Line 7 final elution of LYZ2 (26 kDa) and line 8 is control protein (recombinant nucleocapsid (N) protein of SARS-CoV-2). **Sup 2C:** Western blot analysis of LYZ2 and LYZ2-SH3; line 1, protein size marker; line 2, LYZ2; line 3, LYZ2-SH3b and line 4 is control protein (recombinant nucleocapsid (N) protein of SARS-CoV-2).

**
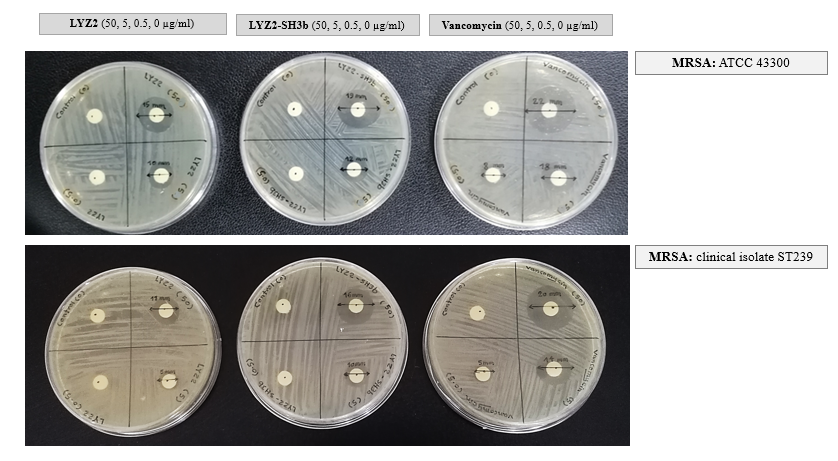
**

**Sup 3**. Disc diffusion diameter for various concentrations (µg/ml) of LYZ2, LYZ2-SH3b, and a control antibiotic (vancomycin) screened against MRSA (ATCC 43300 and clinical isolate ST239). **Abbreviation:** Methicillin-resistant Staphylococcus aureus (MRSA).

**
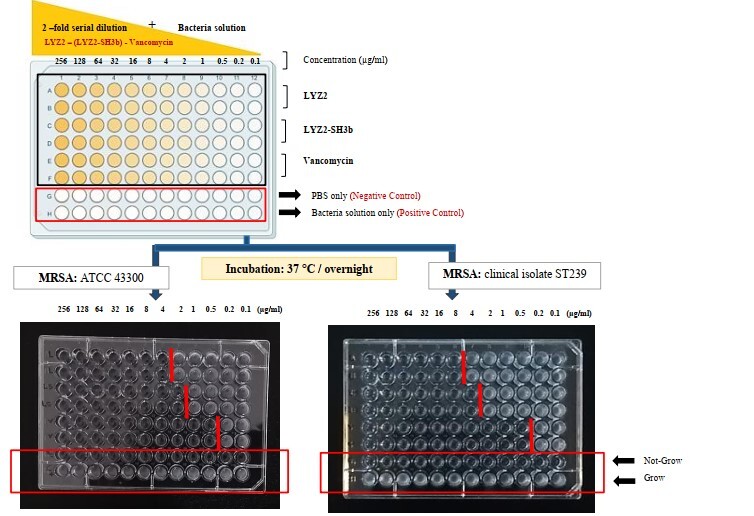
**

**Sup 4:** The minimum inhibitory concentration (MIC in µg//ml) of LYZ2-SH3b and LYZ2 and a control antibiotic (vancomycin) screened against MRSA (ATCC 43300 and clinical isolate ST239). All tests were carried out in duplicate times; **Abbreviation:** Minimal inhibitory concentration (MIC), Methicillin-resistant Staphylococcus aureus (MRSA).


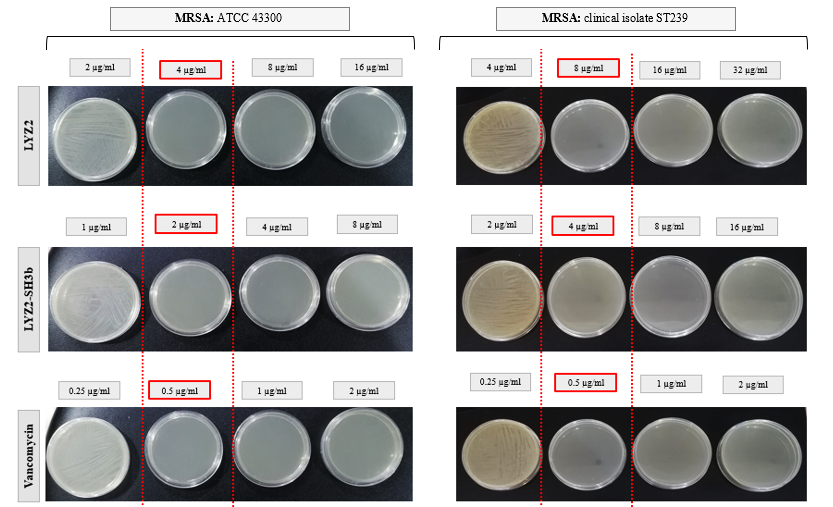


**Sup 5.** The minimum bactericidal concentration (MBC in µg//mL) of LYZ2-SH3b and LYZ2 and a control antibiotic (vancomycin) screened against MRSA (ATCC 43300 and clinical isolate ST239); **Abbreviation:** Minimal bactericidal concentration (MBC), Methicillin-resistant Staphylococcus aureus (MRSA).
